# The Complement System Is Essential for Arteriogenesis by Enhancing Sterile Inflammation as a Relevant Step in Collateral Artery Growth

**DOI:** 10.3390/cells13171405

**Published:** 2024-08-23

**Authors:** Amanda Zhu, Carolin Baur, Philipp Götz, Katharina Elbs, Manuel Lasch, Anna Faro, Klaus T. Preissner, Elisabeth Deindl

**Affiliations:** 1 Institute of Surgical Research at the Walter Brendel Centre of Experimental Medicine, University Hospital, Ludwig-Maximilians-Universität München, 81377 Munich, Germany; amanda.zhu@med.uni-muenchen.de (A.Z.); carolin.baur@med.uni-muenchen.de (C.B.); p.goetz@med.uni-muenchen.de (P.G.); katharina.elbs@med.uni-muenchen.de (K.E.); manuel.lasch@med.uni-muenchen.de (M.L.); anna.braumandl@web.de (A.F.); 2 Biomedical Center, Institute of Cardiovascular Physiology and Pathophysiology, Faculty of Medicine, Ludwig-Maximilians-Universität München, 82152 Planegg-Martinsried, Germany; 3 Department of Otorhinolaryngology, Head and Neck Surgery, University Hospital, Ludwig-Maximilians-Universität München, 81377 Munich, Germany; 4 Department of Cardiology, Kerckhoff-Heart Research Institute, Faculty of Medicine, Justus-Liebig-University, 35392 Giessen, Germany; klaus.t.preissner@biochemie.med.uni-giessen.de

**Keywords:** arteriogenesis, complement C3, macrophages, sterile inflammation, macrophage polarization, mast cells, monocyte chemoattractant protein-1

## Abstract

Arteriogenesis is an inflammatory driven mechanism, describing the growth of a natural bypass from pre-existing collateral arteries to compensate for an occluded artery. The complement system component C3 is a potent natural inflammatory activator. Here, we investigated its impact on the process of collateral artery growth using C3-deficient (C3 −/−) and wildtype control mice in a murine hindlimb model of arteriogenesis. Induction of arteriogenesis by unilateral femoral artery ligation resulted in decreased perfusion recovery in C3 −/− mice on day 7 as shown by Laser Doppler imaging. Immunofluorescence staining revealed a reduced vascular cell proliferation in C3 −/− mice. Gene expression analysis displayed a significant reduction in monocyte chemoattractant protein-1 (MCP-1) expression in C3 −/− mice. Interestingly, 3 days after induction of arteriogenesis, the number of macrophages (CD68^+^) recruited to growing collaterals was not affected by C3 deficiency. However, a significant reduction in inflammatory M1-like polarized macrophages (CD68^+^/MRC1^−^) was noted. Forced mast cell activation by Compound 48/80 as well as exogenous MCP-1 application rescued the number of M1-like polarized macrophages along with perfusion recovery in C3 −/− mice. In summary, this study demonstrates that complement C3 influences arteriogenesis by mediating MCP-1 expression, which is essential for the induction and enhancement of sterile inflammation.

## 1. Introduction

Arterial obstruction such as stenosis or thrombus formation results in significantly reduced perfusion of the distal tissue, leading to ischemia-related damage or even necrosis. Therapeutic options for treating occlusive arterial diseases include interventional techniques, for instance, percutaneous transluminal angioplasty and bypass surgery. However, the body can provide alternative routes to circumvent occluded arteries through vascular adaptation including the growth of collateral arteries. This process is called arteriogenesis and can be explained as the remodeling of pre-existing arteriolar anastomoses to completely developed functional arteries forming a natural bypass [1,2].

In principal, the increased shear stress in pre-existing collateral arteries due to occlusion of a main artery induces arteriogenesis by the following steps: a mechanosensory complex consisting of platelet endothelial cell adhesion molecule-1 (PECAM-1), vascular endothelial cadherin (VE-cadherin) and vascular endothelial growth factor receptor 2 (VEGFR-2) activates the endothelium [3]. As a result, adhesion molecules, such as intercellular adhesion molecule 1 (ICAM-1), are upregulated to allow blood cell adherence at these sites, accompanied by the release of cytokines [4]. In addition, the activated endothelial cells liberate ribonucleic acid (RNA), and this extracellular RNA (eRNA) mediates binding of vascular endothelial growth factor A (VEGF-A) and thereby the activation of VEGFR-2. As a result, von Willebrand factor (vWF) is released from the endothelial cells of growing collateral arteries [5]. Platelets transiently bind to vWF via glycoprotein Ibα (GPIbα), become activated and form aggregates with neutrophils (platelet–neutrophil aggregates, PNA) through interactions between the platelet P-selectin and the P-selectin glycoprotein ligand-1 on leukocytes [6,7]. Interaction with endothelial cells enables the extravasation of leukocytes [6], whereby extravasated neutrophils release reactive oxygen species (ROS) to activate perivascular mast cells. In turn, arteriogenesis is augmented by increasing the bioavailability of cytokines and chemokines, such as tumor necrosis factor-⍺ (TNF-⍺) and monocyte chemoattractant protein-1 (MCP-1) [8]. MCP-1 has a strong chemotactic effect on monocytes and recruits them to the site of inflammation [9]. In the perivascular space, monocytes differentiate into macrophages, which release matrix metalloproteinases (MMPs), cytokines and growth factors, including TNF⍺ and fibroblast growth factor 2 (FGF-2). Together, they promote vascular growth, essential finally for the enlargement of the collateral vessel wall and lumen [10,11,12,13]. 

Macrophages have a high plasticity and are regulated by their local microenvironment in phenotype acquisition along with the respective gene expression and associated functions. A distinction is made between the classically activated or M1-like macrophages, which have a pro-inflammatory effect, and the alternatively activated, pro-regenerative M2-like macrophages [14]. Classically activated macrophages primarily enhance the recruitment of circulating monocytes, while M2-like macrophages mainly promote vascular remodeling and tissue repair [15,16].

Promoting arteriogenesis may present an alternative non-invasive therapeutic approach for patients suffering from vascular occlusive diseases. In order to effectively accelerate arteriogenesis, it is vital to identify the molecular mechanisms underlying the process of collateral artery growth. 

The complement system is a humoral part of the immune system that plays an important role in homeostasis, inflammation and defense against pathogens. It consists of plasma proteins that are mainly produced by the liver, although various other cells also secrete factors of the complement system, forming a proteolytic cascade [17]. Upon increased permeability of the endothelial barrier at the site of inflammation, circulating complement proteins penetrate this cellular region and assist the recruitment of leukocytes as well as the removal of invaded microbes. Under these conditions, the complement cascade can be activated via three different pathways—the classical, the lectin and the alternative pathway—resulting in the cleavage of the central complement component C3 to C3b and C3a. Deposition of C3b on bacterial cell surfaces is critical for their opsonization and is required for subsequent C5 cleavage. The anaphylatoxins C3a and C5a act as potent pro-inflammatory mediators providing strong chemotactic signals in the innate immunity process, while the association of C5b with terminal components C6–C9 is necessary for the formation of the membrane attack complex.

Recently, the complement system has been connected to a variety of pathophysiological processes, such as ischemia–reperfusion injury, autoimmune diseases as well as sepsis [18,19,20,21]. Further studies have shown that complementary components contribute to vascular remodeling processes such as atherosclerosis [22]. In relation to neovascularization, the function of the complement system on angiogenesis has been extensively studied as well, yet providing controversial results: In a model of choroidal neovascularization, C3 deficiency was shown to inhibit angiogenesis, whereas in a model of vascular development in the retina, C3 deficiency led to increased angiogenesis [23,24]. Previous data of our group have shown that the absence of the complement factor C3 enhanced capillary sprouting in ischemic muscle tissue, whereby an elevated number of leukocytes, especially neutrophils, and M2-like macrophages contributed to improved angiogenesis [25]. In general, the complement system plays a decisive role in physiological as well as pathological angiogenesis. Based on the circumstances, however, the complement-dependent mechanisms involved can vary immensely, exhibiting both pro- and antiangiogenic functions (for an overview, see [26]).

Data on the relevance of the complement system in the process of arteriogenesis are scarcely present. In a previous study, we have shown that a single dose of Cobra venom factor (CVF), a C3-like complement-activating protein, led to increased arteriogenesis [27]. Yet, the influence of the complement system, and C3 in particular, on arteriogenesis is still not understood and is subject of the present study.

## 2. Materials and Methods

### 2.1. Animal Protocol and Treatments

All experiments were carried out in strict accordance with the German and NIH animal legislation guidelines and were approved by the Bavarian Animal Care and Use Committee (ethical approval code: ROB-55.2–2532.Vet_02–17–99 and ROB-55.2–2532.Vet_02–22–99).

To investigate the role of C3 in arteriogenesis, 8–12-week-old C3-deficient mice (C3tm1Crr, JAX stock #029661, The Jackson Laboratory, referred here as C3 −/−), bred at the institute, were compared to wildtype (WT) C57BL/6J mice, provided by Charles River Laboratory (Sulzfeld, Germany). 

The mice were treated with daily injections either of bromodeoxyuridine (BrdU, 1.25 mg/day(d) i.p., Sigma-Aldrich, St. Louis, MO, USA), serving as a proliferation marker, Compound 48/80 (C48/80, 0.5 mg/kg/d subcutaneous (s.c.) in the inguinal region, Sigma-Aldrich, C2313–100), a mast cell activator, or monocyte chemoattractant protein-1 (MCP-1, 100 ng/d s.c. in the inguinal region, R&D Systems, Minneapolis, MN, USA) all dissolved in 100 µL phosphate-buffered saline (PBS, 148 mM Na^+^, 1.8 mM K^+^, pH 7.2). Treatments were continued until the end of the experiments.

### 2.2. Experimental Procedures and Tissue Harvesting

Arteriogenesis was induced in male mice by unilateral ligation of the right femoral artery distally to the origin of the profunda femoris branch (femoral artery ligation, FAL), whereas the left leg was sham-operated and served as an internal control, as previously described [28]. 

During the surgical procedures mice were under general anesthesia with a combination of fentanyl (0.05 mg/kg, CuraMED Pharma, Karlsruhe, Germany), midazolam (5.0 mg/kg, Ratiopharm GmbH, Ulm, Germany) and medetomidine (0.5 mg/kg, Pfister Pharma, Berlin, Germany).

Hindlimb perfusion measurements were conducted by Laser Doppler imaging (LDI, Moor LDI 5061 and Moor Software Version 3.01, Moor Instruments, Remagen, Germany) before ligation (baseline), immediately after ligation (aFAL), and 3 days and 7 days after FAL. The body temperature was controlled between 36 and 38 °C, starting 10 min prior to measurements. A flux mean value of a defined region (region of interest, ROI, 0.4 cm^2^) from the ankle to the toes was determined. Tissue perfusion was calculated by flux means of occluded (occ)-to-sham-operated (sham) ratios. 

Superficial collateral arteries were obtained for quantitative real-time PCR (qRT-PCR). For better visualization of the collaterals, a catheter was inserted in the abdominal aorta and both hindlimbs were perfused with latex flexible compound (Spartan Products, Crystal Lake, IL, USA). Two superficial collateral arteries per side were isolated, snap-frozen in dry ice and stored at -80°C until further processing.

For histological analysis, the hindlimb was perfused with adenosine buffer (1% adenosine (Sigma-Aldrich) and 5% bovine serum albumin (BSA, Sigma-Aldrich) dissolved in PBS) to assure maximal vasodilation, followed by 3% paraformaldehyde (PFA, dissolved in PBS, pH 7.4, Merck, Darmstadt, Germany) for fixation of the muscle tissue. The perfusion solutions were warmed up to the murine body temperature to avoid vasoconstriction. Adductor muscles were harvested and kept in a 15% sucrose solution (Sigma-Aldrich) for one hour and a 30% sucrose solution overnight.

### 2.3. Histology and Immunohistology

For histological analyses, the tissue was cryopreserved in Tissue Tek (Sakura Finetek Germany GmbH, Staufen, Germany), and 8 μm thick cross-sections were prepared.

Tissue collected 7 days after induction of arteriogenesis was used to stain for histological analysis of luminal diameter and vascular cell proliferation. Sections were first incubated with 1N HCl for 30 min at 37 °C to denature DNA, subsequently permeabilized with 0.2% Triton X-100 (AppliChem GmbH, Darmstadt, Germany) for 10 min and afterwards labeled with an anti-BrdU antibody (1:50, Abcam, Cambridge, UK, ab6326) at 4 °C overnight. The next day, the secondary antibodies goat anti-rat Alexa Fluor^®^ 546 (1:100, Thermo Fisher, A-11081) and anti-ACTA2-Alexa Fluor^®^ 488 (anti-actin alpha 2, 1:400, Sigma-Aldrich, F3777), a marker for smooth muscle cells in the outer vessel wall, were applied.

To analyze the presence of perivascular macrophages, the tissue was incubated with anti-CD68-Alexa Fluor^®^ 488 (1:200, Abcam, ab201844) and the primary antibody anti-MRC1 (mannose receptor C-type 1, 1:200, Abcam, ab64693) at 4 °C overnight; the secondary antibody donkey anti-rabbit-Alexa Fluor^®^ 546 (1:200, Thermo Fisher, A10040) was added the following day. CD68 stained the macrophages, while MRC1 served as a marker for pro-regenerative M2-like macrophages. The macrophages were counted 3 days and 7 days after FAL.

For all immunofluorescence stainings, sections were incubated with Alexa Fluor^®^ 647 anti-mouse CD31 antibody (1:100, BioLegend, San Diego, CA, USA, 102516) to visualize the endothelial cell layer and 4′,6-diamidino-2-phenylindole (DAPI, 1:1000, Thermo Fisher, 62248) to mark nucleic DNA and finally mounted with Dako mounting medium (Dako, Agilent, Santa Clara, CA, USA).

Giemsa staining was performed according to standard procedures and used to investigate perivascular mast cell accumulation and activation. The mast cells were counted 24 h (h) and 3 days after FAL.

The stained sections were imaged using a Leica DM6 B epifluorescence microscope (Leica microsystems, Wetzlar, Germany) for bright- and dark-field images. The open source software ImageJ2 was used for analysis.

### 2.4. Flow Cytometry Analyses of Whole Blood

Whole blood was collected 24 h after FAL via cardiac puncture and anticoagulated with heparin (10 UE per mL blood, Ratiopharm GmbH). 100 µL of each blood sample was lysed using 2 mL of lysing solution (1:10 in aqua, BD FACS^TM^ Lysing solution, BD Biosciences, Franklin Lakes, NJ, USA, 349202) and afterwards centrifuged (300× *g*, 5 min, RT). Cell pellets were incubated with a solution consisting of 100 µL PBS/1% BSA containing PE anti-mouse CD11b (1:300, BioLegend, 101208), Brilliant Violet 421^TM^ anti-mouse CD115 (1:300, BioLegend, 135513), APC anti-mouse Ly-6G/Ly-6C (1:800, BioLegend, 108412), FITC anti-mouse CD41 (1:400, BioLegend, 135513) and eBioscience^TM^ Fixable Viability Dye eFluor^TM^ 780 (1:1000, Invitrogen, Waltham, MA, USA, 65-0865-14) for 20 min at 4 °C. The samples were washed in PBS and centrifuged. Finally, the pellets were resuspended in 300 µL PBS/1%BSA and analyzed using a BD LSRFortessa^TM^ cell analyzer (BD Biosciences). Gating and analysis of cell populations was conducted using FlowJo V10. 

Whole-blood analyses were performed by generating a differential blood count using the ProCyte Dx (IDEXX laboratories, Westbrook, ME, USA) with mouse-specific settings.

### 2.5. qRT-PCR Analyses

Total RNA was extracted from collateral arteries, isolated 12 h after FAL, according to the single-step method of Chomoczynski and Sacchi [29] using an acid guanidinium thiocyanate–phenol–chloroform mixture. RQ1 RNase-Free DNase (Promega, Madison, WI, USA, M6101) digestion removed residual genomic DNA. A total of 1 µg purified RNA was reverse transcribed into cNDA using the Maxima H Minus cDNA Synthesis Master Mix (Thermo Fisher, M1661). cDNA was diluted 1:5 in RNase-free water and stored at −20 °C until further processing. qRT-PCR was performed with 2 µL diluted cDNA using PowerUp SYBR Green Master Mix (Thermo Fisher, A25742).

Following primers were used: 18S rRNA forward 5′-GGACAGGATTGACAGATTGATAG-3′, 18S rRNA reverse 5′-CTCGTTCGTTATCGGAATTAAC-3′, MCP-1 forward 5′-CTCAAGAGAGAGGTCTGTGCTG-3′, and MCP-1 reverse 5′-GTAGTGGATGCATTAGCTTCAG-3′. The protocol was performed in a StepOnePlus cycler (Applied Biosystems, Carlsbad, CA, USA): initial denaturation (95 °C for 2 min) was followed by 40 cycles of denaturation (95 °C for 15 s), annealing (64 °C for 1 min) and extension (60 °C for 1 min). Three independent qRT-PCR reactions were performed on each template. Specific amplification was ensured using melt curve analyses and agarose gels. The results were normalized to the expression levels of the 18S rRNA. 

### 2.6. Statistical Analyses

The results were analyzed with GraphPad Prism 10 (GraphPad Software, LA Jolla, CA, USA). Comparisons between groups were calculated as stated in figure legends.

All data are presented as mean ± standard error of the mean (SEM). The results are considered statistically significant at *p* < 0.05.

## 3. Results

### 3.1. The Absence of C3 in Mice Reduces Perfusion Recovery in Arteriogenesis

To investigate whether the complement system has an impact on arteriogenesis, we employed a murine hindlimb model, in which femoral artery ligation (FAL) results in collateral artery growth [28]. 

#### 3.1.1. Impact of C3 Deficiency on Perfusion Recovery

**Figure 1:**
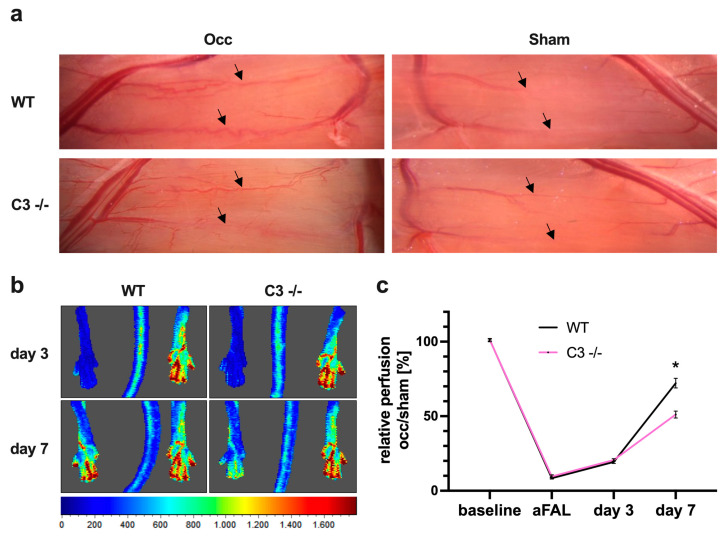
C3 −/− mice show reduced perfusion recovery after femoral artery ligation (FAL). (**a**) Representative photographs of superficial collateral arteries (arrows) in the adductor muscles of wildtype (WT, upper panels) and C3 −/− (lower panels) mice. Left panels: Grown collateral arteries of the occluded leg (Occ) have increased caliber size and display a corkscrew-like pattern. Right panels: Pre-existing collaterals on the sham-operated leg (Sham) appear as thin linear vessels. Photographs were taken 7 days after FAL. (**b**) Representative flux images of Laser Doppler measurements of wildtype (WT, left images) and C3 −/− mice (right images) 3 days and 7 days after FAL. Flux scale bar shown below (red = high flow, blue = low flow). (**c**) Line graph displaying the relative perfusion (Occ/Sham) of WT and C3 −/− mice before FAL (baseline), directly after FAL (aFAL), at day 3 and day 7 after FAL. Data shown are means ± SEM, *n* = 5 mice per group, * *p* < 0.05 WT compared to C3 −/− by two-way ANOVA with Bonferroni’s multiple comparison test.

Compared to wildtype (WT) control mice, C3 −/− mice showed a reduced increase in caliber size and tortuosity of superficial collateral arteries 7 days after the surgical procedure (Figure 1a). Moreover, Laser Doppler measurements demonstrated a significant reduction in perfusion recovery (Figure 1b,c). 

#### 3.1.2. Impact of C3 Deficiency on Vascular Cell Proliferation

**Figure 2:**
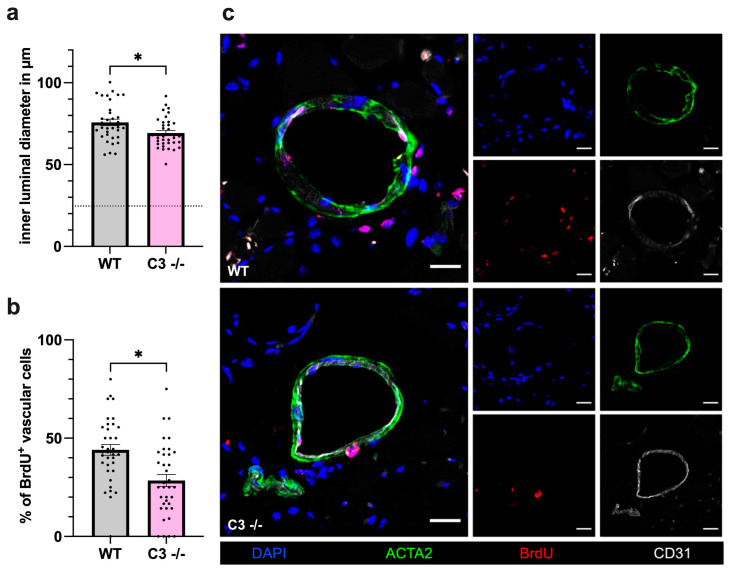
C3 deficiency reduces vascular cell proliferation. The scatter plots with bars show (**a**) the inner luminal diameter of growing collaterals and (**b**) the percentage of proliferating vascular cells (BrdU^+^ cells) per growing collateral of wildtype (WT) and C3 −/− mice 7 days after femoral artery ligation (FAL). The dashed horizontal line in (**a**) represents the mean sham value. Data shown are means ± SEM, *n* = 6 mice per group with 3 slices, 2 collaterals each, * *p* < 0.05 WT compared to C3 −/− by unpaired student’s *t*-test. (**c**) Representative images of immunofluorescence staining of growing collateral arteries showing merged images (left) and single stain (right) of WT (upper panels) and C3 −/− (lower panels) mice 7 days after FAL. BrdU (red) served as a proliferation marker, ACTA2 (green) served as a smooth muscle cell marker, CD31 (white) visualized the endothelial cell layer and DAPI (blue) labeled nuclei. Scale bar: 20 µm.

To confirm that the reduced perfusion recovery observed in C3 −/− mice was due to decreased collateral artery growth, we performed immunofluorescence analyses of the adductor muscle collected 7 days after FAL. CD31 was used as a marker for endothelial cells, the inner vascular layer, in order to analyze the inner luminal diameter. Compared to WT mice, C3 −/− mice showed a significantly limited increase in the luminal diameter after FAL (Figure 2a). To investigate whether the reduced luminal diameter in C3 −/− mice was due to reduced vascular cell proliferation and not due to reduced vasodilation, tissue staining for bromodeoxyuridine (BrdU) as a cell proliferation marker was performed. Here, the smooth muscle cell marker ACTA2 was applied to stain the outer vessel wall, and BrdU^+^ cells within the vascular structure were defined as proliferating vascular cells. The histological analyses revealed a significantly lower percentage of proliferating vascular cells in C3 −/− mice compared to WT controls (Figure 2b,c). The evaluation of the sham-operated side revealed no differences in either luminal diameter or vascular cell proliferation between the two groups.

Taken together, these results indicate a crucial role for the complement system, especially C3, in the process of arteriogenesis. 

### 3.2. The Absence of C3 Leads to Diminished Macrophage Recruitment and Alters Macrophage Polarization during Arteriogenesis in Mice

**Figure 3:**
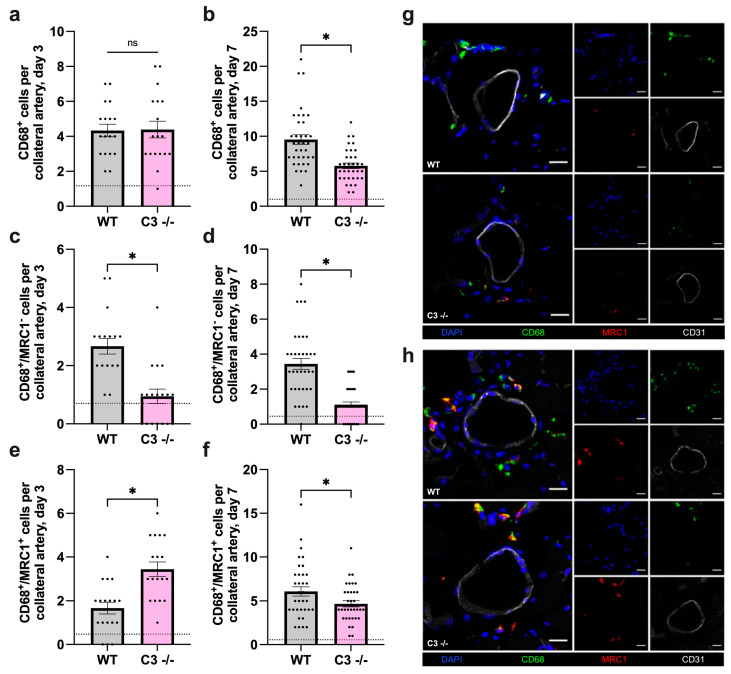
C3 deficiency impairs M1-like macrophage polarization after induction of arteriogenesis. The scatter plots with bars display (**a**) the total number of perivascular macrophages (CD68^+^ cells) 3 days and (**b**) 7 days after femoral artery ligation (FAL), the number of M1-like macrophages (CD68^+^/MRC1^−^ cells) (**c**) 3 days and (**d**) 7 days after FAL, as well as (**e**) the number of M2-like macrophages (CD68^+^/MRC1^+^ cells) per collateral artery on day 3 and (**f**) day 7 after induction of arteriogenesis of WT and C3 −/− mice. The dashed horizontal line represents the mean sham value. Data shown are means ± SEM, *n* = 3 mice per group with 3 slices, 2 collaterals each on day 3, and *n* = 6 mice per group with 3 slices, 2 collaterals each on day 7 after FAL, * *p* < 0.05 and ns ≥ 0.05 WT compared to C3 −/− by unpaired student’s *t*-test. (**g**) Representative images of immunofluorescence stains of growing collateral arteries of WT (upper panel) and C3 −/− (lower panel) mice 3 days and (**h**) 7 days after FAL. CD68 (green) served as a marker for macrophages, MRC1 (red) served as a marker for M2-like macrophages, CD31 (white) labeled the endothelial cell layer and DAPI (blue) labeled nuclei. Scale bar: 20 µm.

During the process of arteriogenesis, macrophages and their polarization state play an important role in vascular remodeling and growth. To analyze perivascular macrophage accumulation, 3 and 7 days after induction of arteriogenesis, adductor muscle tissue was harvested to detect infiltrated macrophages by immunofluorescence staining (Figure 3a–h). 

#### 3.2.1. Impact of C3 Deficiency on Macrophage Recruitment and Polarization 3 Days after FAL

We found that the number of CD68^+^ cells, and thus the extent of macrophage recruitment, did not differ between WT and C3 −/− mice at day 3 (Figure 3a). However, the number of M1-like macrophages (CD68^+^/MRC1^−^) was significantly lower in C3 −/− mice compared to WT controls (Figure 3c), while the number of M2-like polarized macrophages (CD68^+^/MRC1^+^) was significantly increased (Figure 3e). Previous studies have shown that, in the process of arteriogenesis, there is a strong increase in M1-like macrophages on day 3 after FAL [16], suggesting a major role of complement factor C3 in the generation of M1-like polarized macrophages.

#### 3.2.2. Impact of C3 Deficiency on Macrophage Recruitment and Polarization 7 Days after FAL

Upon analysis of adductor muscles, harvested 7 days after induction of arteriogenesis, a significantly decreased number of perivascular macrophages per growing collateral artery was noted in C3 −/− mice compared to WT mice (Figure 3b). The observed reduction in macrophage accumulation was mainly due to a significantly reduced number of M1-like macrophages (CD68^+^/MRC1^−^) (Figure 3d), although the number of M2-like macrophages (CD68^+^/MRC1^+^) was also affected, albeit to a lesser extent (Figure 3f). Taken together, these data indicate that the complement system plays a decisive role in the recruitment of macrophages and their polarization, particularly to inflammatory M1-like cells.

### 3.3. The Absence of C3 Reveals No Impact on Platelet–Leukocyte Aggregate Formation and Perivascular Mast Cell Activation in Arteriogenesis in Mice

Mast cells contribute to arteriogenesis via recruitment of T-cells, neutrophils and monocytes, as well as through growth factor and cytokine release [8]. They can regulate the local microenvironment of macrophages, including the liberation of complement factors [30]. We have previously shown that mast cell activation in the perivascular space of collaterals is mediated by platelet activation via platelet receptor GPIb⍺, followed by platelet–leukocyte aggregate (PLA) formation [6]. In addition to platelet–neutrophil aggregates (PNA), the formation of monocyte–platelet aggregates (MPA) also serves as a sensitive marker for activated platelets [31]. To examine whether the reduced number of M1-like macrophages, particularly in the early phase of arteriogenesis (day 3), was due to diminished PNA formation or mast cell activation, flow cytometry and histological analyses were performed. 

#### 3.3.1. Impact of C3 Deficiency on Platelet–Leukocyte Aggregate Formation

Following the analysis of the number of PLA as well as the platelet, neutrophil and monocyte counts 24 h after FAL in blood samples of C3 −/− and WT mice, no differences in either PNA and MPA formation or in the number of peripheral blood platelets, neutrophils and monocytes were noted between C3 −/− and WT mice (Supplementary Materials, Figure S1a–h).

#### 3.3.2. Impact of C3 Deficiency on Mast Cell Recruitment and Activation

Using Giemsa staining, the total number as well as the number of degranulated mast cells in the perivascular space of growing collaterals were analyzed 24 h and 3 days after FAL. Again, no difference in the extent of mast cell recruitment and activation was seen between C3 −/− and WT mice, either 24 h or 3 days after induction of arteriogenesis (Supplementary Materials, Figure S2a–e). However, the analyses showed that FAL resulted in a significantly elevated number of degranulated mast cells compared to the sham-operated side in each mouse strain (WT occ: 0.63 ± 0.1 vs. WT sham: 0.22 ± 0.09; C3 −/− occ: 0.65 ± 0.09 vs. C3 −/− sham: 0.22 ± 0.09, *p* < 0.05, unpaired student’s *t*-test).

These data indicate that the diminished number of M1-like macrophages in C3 −/− mice is not due to impaired platelet activation, PLA formation and subsequent mast cell activation in the process of arterial remodeling.

### 3.4. The Absence of C3 Interferes with MCP-1 Expression in Growing Collateral Arteries in Mice

In the process of arteriogenesis, MCP-1 has been reported to be of major relevance for leukocyte recruitment, especially for monocytes and macrophages [8,32]. Moreover, MCP-1 has been described to induce M1-like macrophage polarization [33]. We have previously shown that MCP-1 expression increases in growing collateral arteries due to shear stress induction after FAL compared to the sham-operated side [8]. 

#### 3.4.1. Impact of C3 Deficiency on MCP-1 Expression

**Figure 4:**
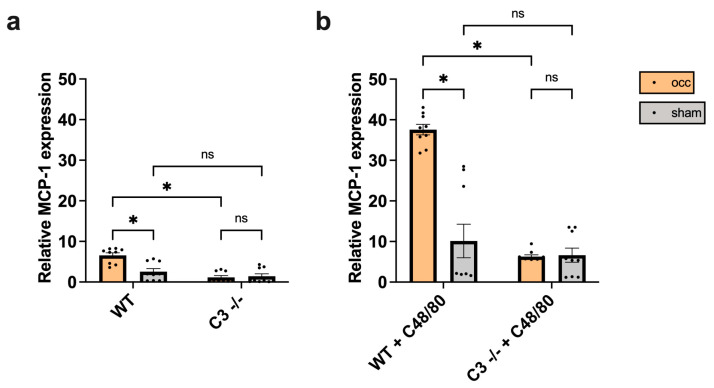
C3 is essential for MCP-1 expression in arteriogenesis. The scatter plots with bars show (**a**) the mRNA expression level of MCP-1 in collaterals of untreated wildtype (WT) and C3 −/− mice as well as (**b**) WT and C3 −/− mice treated with C48/80, as quantified by qRT-PCR 12 h after femoral artery occlusion (occ, orange bars) or sham operation (sham, grey bars). The data were normalized to the expression level of the 18S rRNA and shown are means ± SEM, *n* = 3 in triplicates per group, * *p* < 0.05 and ns ≥ 0.05, compared by two-way ANOVA.

To examine whether the reduced number of M1-like macrophages could be a result of impaired MCP-1 expression in C3 −/− mice, qRT-PCR analysis was performed on collaterals isolated from the occluded and the sham side of WT and C3 −/− mice 12 h after FAL. Indeed, the analyses revealed that the increase in MCP-1 expression on the occluded side is only observed in WT but not in C3 −/− mice, which in general exhibited a very low MCP-1 mRNA expression level (Figure 4a).

#### 3.4.2. Impact of C48/80 on MCP-1 Expression in C3 −/− Mice

Previous data from our group have shown that targeted mast cell activation enhances the expression level of MCP-1 in the process of arteriogenesis [8]. Accordingly, following treatment with the mast cell activator C48/80, collaterals were isolated 12 h after FAL from the occluded and the sham side of WT and C3 −/− mice. qRT-PCR analyses evidenced that the levels of MCP-1 mRNA in C3 −/− mice could be rescued using targeted mast cell activation by C48/80 and brought to the level of untreated WT mice (untreated WT mice occ: 6.57 ± 0.63 vs. C3 −/− mice + C48/80 occ: 6.26 ± 0.46, unpaired student’s *t*-test, not significant), while WT mice treated with C48/80 showed a significant increase in MCP-1 expression in growing versus resting (sham site) collaterals (Figure 4b). Regarding mast cell activation (using Giemsa stain), our results revealed that both WT and C3 −/− mice equally responded to C48/80 exposure (Supplementary Materials, Figure S3a–d). These data indicate that the bioavailability of MCP-1 in growing collateral arteries is augmented by complement factor C3.

### 3.5. Forced Mast Cell Activation Rescues Perfusion Recovery in C3 −/− Mice via Promotion of MCP-1 Expression and Subsequent M1-Like Macrophage Recruitment

To analyze whether forced mast cell activation may provide the necessary MCP-1 expression for M1-like macrophage recruitment, independently of the complement system, C3 −/− mice were treated with C48/80. In parallel, another group of C3 −/− mice was treated with recombinant mouse MCP-1 in order to confirm that the results are mainly due to increased MCP-1 expression.

#### 3.5.1. Impact of C48/80 or MCP-1 on Macrophage Recruitment and Polarization in C3 −/− Mice

**Figure 5:**
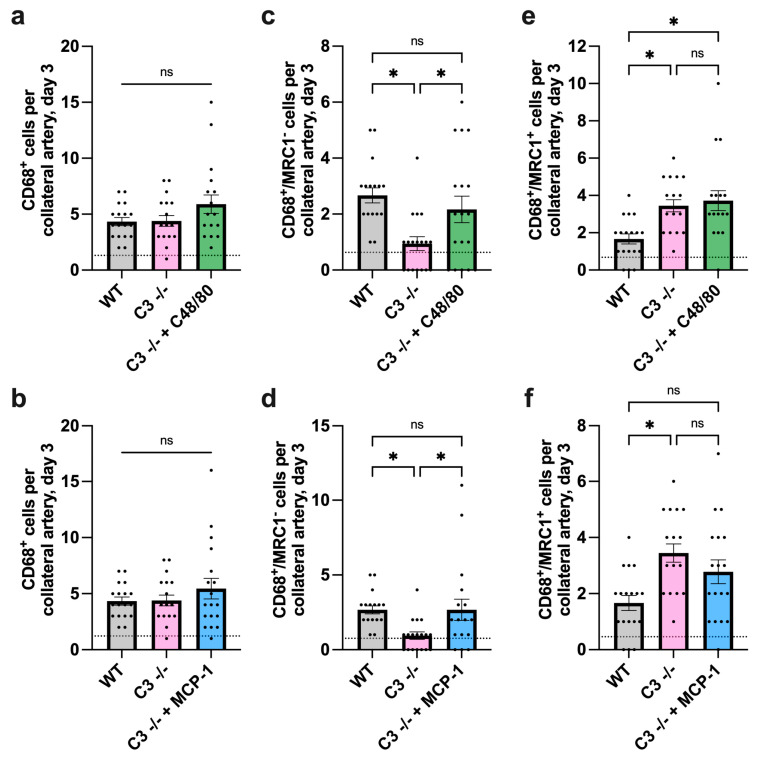
Additional MCP-1 availability increases the number of M1-like macrophages in C3 −/− mice 3 days after induction of arteriogenesis. Scatter plots with bars representing (**a**) the total number of perivascular macrophages (CD68^+^ cells) of wildtype (WT, grey bars), C3 −/− (pink bars) and C3 −/− after treatment with C48/80 (green bars) or (**b**) MCP-1 (blue bars), the number of M1-like macrophages (CD68^+^/MRC1^−^ cells) after treatment with (**c**) C48/80 or (**d**) MCP-1 as well as the number of M2-like macrophages (CD68^+^/MRC1^+^ cells) after treatment with (**e**) C48/80 or (**f**) MCP-1 per collateral artery on day 3 after femoral artery ligation (FAL). The dashed horizontal line represents the mean sham value. Data shown are means ± SEM, *n* = 3 mice per group with 3 slices, 2 collaterals each, * *p* < 0.05 and ns ≥ 0.05, compared by one-way ANOVA with Bonferroni’s multiple comparison test.

Histological analyses revealed that treatment with the mast cell activator C48/80 as well as with MCP-1 showed little difference in the total number of recruited macrophages 3 days after induction of arteriogenesis (Figure 5a,b). However, treatment with C48/80 or MCP-1, respectively, resulted in an increased recruitment of M1-like polarized macrophages in C3 −/− mice and could thus be brought to the level of untreated WT mice (Figure 5c,d), while it did not influence the number of M2-like macrophages in C3 −/− mice 3 days after FAL (Figure 5e,f). 

Furthermore, 7 days after FAL, the total number of macrophages drastically increased in C3 −/− mice treated with C48/80, whereas treatment with MCP-1 merely brought the total number of macrophages to the level as observed in untreated WT mice (Supplementary Materials, Figure S4a,b). While treatment with MCP-1 rescued C3 −/− mice particularly by augmenting the number of M1-like macrophages to the level of WT mice, both M1- and M2-like macrophages increased through treatment with C48/80 even compared to WT mice (Supplementary Materials, Figure S4c–f).

#### 3.5.2. Impact of C48/80 or MCP-1 on Vascular Cell Proliferation and Perfusion Recovery in C3 −/− Mice

**Figure 6:**
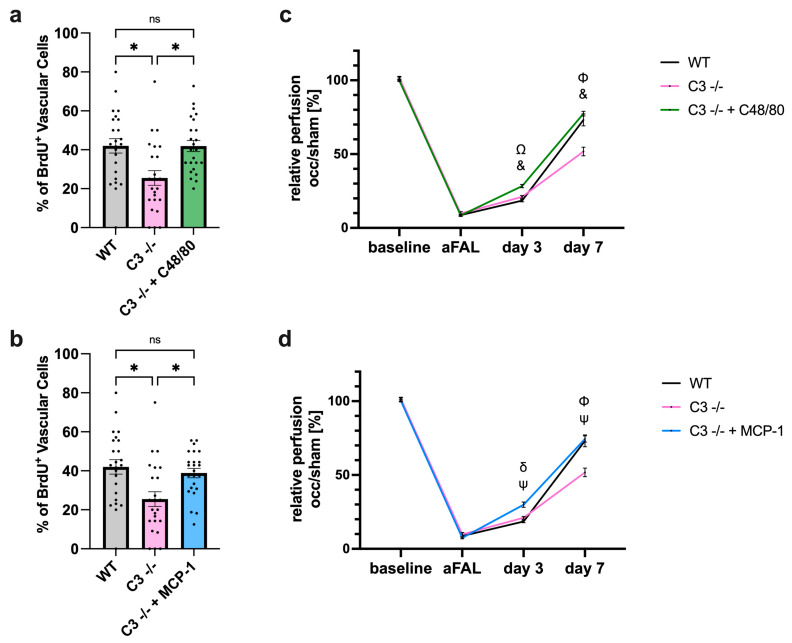
Treatment with C48/80 as well as with MCP-1 rescues arteriogenesis in C3 −/− mice. Scatter plots with bars show the percentage of proliferating vascular cells (BrdU^+^ cells) in WT, C3 −/− and C3 −/− treated with (**a**) C48/80 or (**b**) MCP-1 collateral arteries 7 days after FAL. *n* = 4 mice per group, 3 slices with 2 collaterals each were analyzed per mouse. * *p* < 0.05 and ns ≥ 0.05, one-way ANOVA with Bonferroni’s multiple comparison test. Line graphs displaying the perfusion ratio (occ/sham) before femoral artery ligation (FAL, baseline), directly after FAL (aFAL), at day 3 and day 7 in wildtype (WT), C3 −/−, and C3 −/− mice after treatment with (**c**) C48/80 or (**d**) MCP-1 as measured by LDI. *n* = 4 mice per group. ^Φ^ *p* < 0.05 (WT compared with C3 −/−), ^Ω^ *p* < 0.05 (WT compared with C3 −/− + C48/80), ^&^ *p* < 0.05 (C3 −/− compared with C3 −/− + C48/80), ^δ^ *p* < 0.05 (WT compared with C3 −/− + MCP-1), ^ψ^ *p* < 0.05 (C3 −/− compared to C3 −/− + MCP-1), two-way ANOVA with Bonferroni’s multiple comparison test. Data are means ± SEM.

The exposition towards C48/80 as well as the stimulation with MCP-1 significantly increased vascular cell proliferation in C3 −/− mice to the level of WT mice (Figure 6a,b). Accordingly, perfusion recovery was rescued in treated C3 −/− mice 7 days after FAL (Figure 6c,d).

Taken together, these data indicate that the effect of complement C3 on perivascular macrophage accumulation and polarization towards inflammatory M1-like macrophages is mediated by augmented MCP-1 expression and is therefore necessary to sustain sufficient sterile inflammation for effective collateral artery growth.

## 4. Discussion

In this study, we demonstrate that several steps in arteriogenesis are significantly impaired in mice deficient in complement factor C3, mostly due to reduced MCP-1 expression and limited perivascular accumulation of M1-like macrophages in collateral vessels. 

To investigate the impact of the complement factor C3 on arteriogenesis, we used a murine hind limb model of collateral artery growth to compare various steps of collateral formation in C3 −/− vs. WT mice. Our results show that the blockade of the complement cascade downstream of C3 has a negative impact on the growth of collateral arteries and results in significantly poorer vessel perfusion than in WT mice. The histological findings revealed a reduced luminal diameter along with reduced proliferation of vascular cells in C3 −/− mice supporting the proposed mechanism of arteriogenesis as a combination of steps as seen in innate immunity. In this context, many studies have documented that C3 is an important mediator in inflammatory processes. For instance, C3 expression has been shown to correlate with the severity of renal fibrosis, while C3 deficiency reduced the recruitment of inflammatory cells and pro-inflammatory cytokine production [34].

Furthermore, treatment with CR2-Crry, a specific inhibitor of C3 activation, has been demonstrated to protect against ethanol-induced hepatic steatosis and inflammation [35]. On the other hand, complement factor C3 also has protective effects. In a mouse model of chronic obstructive pulmonary disease, C3 presented anti-oxidative effects as an important pro-survival molecule in airway epithelial cells [36]. In conjunction with our results, this indicates that complement factor C3 or its downstream activation fragments, such as C3a, C5a and C5b-9, is a necessary mediator for the creation of the inflammatory environment in the process of arteriogenesis. It is of note that other mechanisms can substitute for the C3-dependent C5 convertase, leading to the generation of biologically active C5a [37,38,39]. However, the inhibition of the complement cascade at C3 results in a lack of systemic activation of C5 to C5a and C5b.

Several studies have already established the importance of macrophages in arteriogenesis [10,12,16]. The release of growth factors, cytokines and matrix metalloproteases is essential for cell proliferation and remodeling processes, and their number in the perivascular space directly correlates with collateral artery growth [12]. While in a previous study, a strong increase in classically activated M1-like macrophages was found on day 3 after induction of arteriogenesis [16], here, a reduced number of perivascular inflammatory M1-like macrophages were seen in C3 −/− mice as compared to WT mice. Yet, the total number of CD68^+^ cells between C3 −/− and WT mice did not differ. These results coincide with data obtained by Ruan et al. in a mouse model of deoxycorticosterone acetate salt-induced hypertension, where C3 deficiency resulted in the reduction in M1-like macrophages in the perivascular adipose tissue. Furthermore, C3 mRNA expression was found to be decreased in M2-like macrophages but increased in M1-like macrophages, critical for macrophage polarization toward the M1 phenotype by promoting nuclear factor kappa B-dependent transcriptional activity [40]. Their conclusion that complement component C5a regulates cell polarization with macrophages as the main source of complement in this mechanism is likely to apply to our findings. However, it is probable that complement components in turn regulate other pro-inflammatory stimuli in the process of arteriogenesis that are responsible for the perivascular accumulation of M1-like macrophages. Macrophage polarization is the result of a combination of external signals that macrophages receive from their environment. Even within the same location and population, macrophages can exhibit different and constantly changing phenotypes, so that they represent an extremely plastic cell population [41]. In addition to the classically activated M1 macrophages, several other activation types, including the M2 macrophages, have been described. It is important to note that the M1/M2 nomenclature merely reflects the extreme limits of possible differentiation [42]. Accordingly, in this study we refer to CD68^+^/MRC1^−^ macrophages as M1-like macrophages, with pro-inflammatory properties, as opposed to CD68^+^/MRC1^+^ cells as M2-like macrophages, which display anti-inflammatory, regenerative features. The shift from M1-like to M2-like macrophages in an inflammatory process allows these cells to perform different tasks in the different phases of the inflammatory response. With regard to their biological functions of releasing inflammatory and chemotactic cytokines, such as TNF-⍺, MCP-1 or iNOS [43,44,45,46], M1-like macrophages led to an increased recruitment of monocytes during collateral artery growth. Indeed, our analyses identified a reduced number of perivascular macrophages (CD68^+^ cells) on day 7 after induction of arteriogenesis in C3 −/− mice compared to WT mice. Still, the number of M1-like macrophages was reduced in C3 −/− mice, whereas the predominant pool of macrophages consisted of M2-like macrophages. Inflammatory cytokines of M1-like macrophages such as IL-1⍺ and IL-6 have been described to lead to migration and proliferation of vascular smooth muscle cells [47,48,49], suggesting that M1-like macrophages (in WT mice) also play a relevant role in modulating the growth of vascular cells. Together with the abovementioned functions of macrophages, our data indicate that the reduced number of M1-like macrophages contributed to the impaired collateral artery growth in C3 −/− mice, due to a response of complement components downstream of C3 to macrophage polarization in the process of collateral artery growth. 

Mast cells indirectly promote arteriogenesis by augmenting the inflammatory reaction, and their activation is known to be a prerequisite for perivascular macrophage recruitment in the process of arteriogenesis. Mast cell activation in the perivascular space is mediated, among other things, by platelet activation, PNA formation and subsequent ROS formation in neutrophils [8]. The current study indicates that PNA formation as well as subsequent mast cell activation in experimental arteriogenesis are not affected by the absence of complement factor C3 and are therefore not decisive for the reduced number of M1-like macrophages.

It has been shown that MCP-1 supports vessel growth through monocyte recruitment, which adhere to the endothelium and extravasate into the perivascular space [12,32]. Furthermore, numerous reports have described that MCP-1 can polarize macrophages to the pro-inflammatory M1-like phenotype [33,50,51]. In our model, we have previously shown that FAL has led to increased levels of MCP-1 mRNA in growing collaterals due to vascular cell activation through shear stress [8]. However, C3 −/− mice failed to display an increased expression of MCP-1 after FAL, in contrast to a significant increase in this cytokine at the occluded side in WT mice. Consistent with our previous data demonstrating that mast cell degranulation resulted in an increase in MCP-1 expression in the process of collateral artery growth [8], the levels of MCP-1 mRNA in C3 −/− mice could be rescued by forced mast cell degranulation using C48/80. WT mice treated with C48/80 displayed a significant increase in MCP-1 expression as well. Our findings show that the expression of MCP-1 on the sham-operated side was very small when compared to the occluded side of untreated or C48/80-treated mice. Since vascular cells on the sham-operated side were not activated through shear stress, there is no major increase in MCP-1 expression apart from additional mast cell degranulation. Although the contribution of MCP-1 is only one step in the process of arteriogenesis, without its induction through shear stress no collateral artery growth can be observed via C48/80 administration itself.

Various studies have described a relationship between the expression of MCP-1 and the complement system: For instance, both C3a and C5a have been found to significantly induce MCP-1 production in human mast cells [52]. Furthermore, human dermal microvascular endothelial cells have been shown to constitutively express receptors for C3a and C5a. While C5a also enhanced MCP-1 production in a dose-dependent manner, endothelial MCP-1 levels were not changed under the treatment with C3a [53]. Another study using mouse dermal microvascular endothelial cells reported that C5a by itself did not alter chemokine production, whereas the sequential addition of IL-6, IFN-y or LPS followed by C5a revealed enhanced secretion of MCP-1. The authors hypothesized that IL-6, which is released into the plasma at an early stage during an acute inflammation, induced cellular changes that caused the endothelium to become hyper-responsive towards C5a [54]. In context with these results, our observations that MCP-1 mRNA levels in C3 −/− mice were low on the sham side and did not increase after femoral artery occlusion indicate that the complement system downstream of C3 is necessary for adequate MCP-1 expression in general as well as for the shear stress-mediated increase in MCP-1 mRNA expression level in the process of arteriogenesis. In particular investigations on C5a receptor (C5aR)-deficient mice would be interesting to define the role of the C3 downstream complement factor C5a in MCP-1 production in the process of arteriogenesis. 

Complement activation, together with Toll-like receptors, has been shown to promote the differentiation of T-helper 17 (Th17) cells [55], which are known for their role in inflammation and autoimmune diseases. IL-17, a cytokine produced by Th17 cells, has been described to upregulate the expression of MCP-1 [56]. Moreover, another study proposed that Th17-derived IL-17 promotes neovascularization by enhancing inflammation and the production of angiogenic cytokines [57]. Together, these findings suggest that complement-dependent Th17 cell differentiation could potentially play a significant role in arteriogenesis by influencing MCP-1 expression via their cytokine production. Since the role of T cells and particularly Th17 cells is not clarified in detail for arteriogenesis, this topic might be of major interest for further studies.

Another aspect is the IL-31/IL-33 axis, which plays a role in various immune responses including inflammation and tissue remodeling [58,59]. IL-33 has been observed to induce the production and release of MCP-1 [60]. However, it is not yet clear whether the complement system is involved in this process and could therefore be a new interesting angle, especially in the research of arteriogenesis. MCP-1 production occurs in various cells, including mast cells, macrophages or endothelial cells [61]. Even smooth muscle cells have been described to be an important source of MCP-1 in vivo [62]. The major source of MCP-1 in the process of arteriogenesis, nevertheless, is subject for further studies. 

The microbiome plays a significant role in the development of atherosclerotic cardiovascular disease (ASCVD), particularly through gut metabolites like trimethylamine N-oxide (TMAO) [63]. TMAO is a pro-atherosclerotic molecule, with ASCVD patients showing significantly higher levels of TMAO compared to healthy individuals [64]. TMAO promotes atherosclerosis by increasing macrophage migration and foam cell formation in cholesterol plaques, as well as contributing to endothelial dysfunction and vascular inflammation by recruiting leukocytes to endothelial cells [65,66]. Additionally, the composition of microbiota regulates C3 expression in both healthy humans and mice [67]. Vitamin D has been observed to play a role in modulating the immune system, including activating the complement system, enhancing immune responses and exerting anti-inflammatory effects [68,69]. Furthermore, it has been shown to exert protective effects on atherosclerosis, such as the differentiation of vascular smooth muscle cells and improved endothelial function [70]. While atherosclerosis results in adverse vascular inward remodeling, arteriogenesis leads to positive outward remodeling cumulating in the formation of a natural bypass. Despite these differences, mechanisms that contribute to atherosclerosis are also involved in arteriogenesis as both processes depend on immune cells. Together these facts suggest that the microbiome as well as Vitamin D may also have an influence on arteriogenesis. However, further studies are necessary to elucidate possible relationships in detail.

Targeted mast cell activation by C48/80 did not lead to the same increase in MCP-1 expression in C3 −/− mice as in WT mice, indicating that the additional enhancement in MCP-1 mRNA levels in collateral arteries is also dependent on the complement system downstream of C3. Regarding the influence of MCP-1 on macrophage polarization described in the literature; this suggests that the reduced number of M1-like macrophages detected in C3 −/− mice is a result of a missing augmentation of MCP-1 bioavailability in the process of experimental arteriogenesis. 

Treatment with C48/80 or MCP-1 indeed elevated the number of M1-like macrophages to the level of untreated WT mice 3 days after induction of arteriogenesis. Interestingly, only a shift in macrophage polarization towards the M1-like phenotype, but not an increase in the total number of macrophages, was observed in both treatment groups. Seven days after FAL, MCP-1 application restored the number of M1-like macrophages and thus the total number of perivascular macrophages in C3 −/− mice to the level of untreated WT mice. The exposition towards C48/80 led to an increase in both M1-like and M2-like macrophages in C3 −/− mice, not only rescuing the number of M1-like macrophages but significantly increasing the total number of macrophages compared to untreated WT mice. The excessive effect on macrophage recruitment through C48/80 might not be restricted to the activity of MCP-1 since the specific influence of C48/80 on mast cells in arteriogenesis leads to the release of a large variety of cytokines and chemokines [8]. In our current study, administration of C48/80 or MCP-1 restored perfusion recovery in C3 −/− mice, corroborating the relationship between the complement system and MCP-1 in arteriogenesis. Accordingly, we found that treatment with C48/80 as well as MCP-1 significantly improved vascular cell proliferation in C3 −/− mice.

The role of MCP-1 in regulating macrophage recruitment and polarization has a significant impact in vascular biology, influencing atherosclerosis, angiogenesis and arteriogenesis. Similar to our findings for the process of arteriogenesis, MCP-1 expression is induced in the early phase of atherosclerosis leading to the migration of monocytes to the sites of endothelial injury or dysfunction [71]. Elevated MCP-1 levels are associated with increased plaque size and instability and a relatively large numbers of pro-inflammatory M1-like macrophages in plaques [71,72,73]. The absence of MCP-1 or its receptor CCR2 conversely results in a significant reduction in arterial lipid deposition [9], stressing MCP-1 as a driving force in the development of atherosclerosis. 

MCP-1 is also well known for its function in mediating the migration and infiltration of leukocytes in the process of angiogenesis [61,74,75]. Comparable to arteriogenesis, in angiogenesis macrophages and neutrophils provide growth factors and are involved in endothelial cell activation and matrix remodeling [76,77]. Interestingly, it has been observed that a lack of M1-like macrophages results in impaired angiogenesis [78], just as we observed in this study that a reduced number of M1-like macrophages leads to reduced collateral vessel growth. Overall, these findings show the relevance of MCP-1 in pathological and reparative processes and underline the importance of early MCP-1 expression and its influence on macrophage recruitment and polarization to the pro-inflammatory M1-like phenotype. 

Looking at differences in genetic background, environmental and lifestyle factors that cannot be fully accounted for in animal studies as well as drug metabolism and efficacy between mice and humans are only some examples of limitations of investigations in animal models. A further limitation of our study is the use of a knockout model. Other relevant functions of the complement system might have been compensated for to avoid severe or even life-threatening consequences and may thus be still undiscovered. 

In summary, we identified the complement system downstream of C3 as a necessary mediator for the induction and enhancement of sterile inflammation in association with arteriogenesis. Complement-mediated increased bioavailability of MCP-1 is required for sufficient inflammatory M1-like macrophage recruitment in order to promote collateral artery growth.

## Data Availability

The data presented in this study are available on request from the first author.

## Supplementary Materials

The following supporting information can be downloaded at https://www.mdpi.com/article/10.3390/cells13171405/s1.
