# Supplementary material for: The Complement System Is Essential for Arteriogenesis by Enhancing Sterile Inflammation as a Relevant Step in Collateral Artery Growth
Source: Cells. 2024 Aug 23;13(17):1405. doi: 10.3390/cells13171405 (PMC11394660; doi:10.3390/cells13171405)
Supplement: Supplementary file 1 [file cells-13-01405-s001.zip › cells-3150273-supplementary.pdf]

cells

MDPI

Article

The Complement System is Essential for Arteriogenesis by Enhancing Sterile Inflammation as a Relevant Step in Collateral Artery Growth

Amanda Zhu [1,2], Carolin Baur [1,2], Philipp Götz [1,2], Katharina Elbs [1,2], Manuel Lasch [1,2,3], Anna Faro [1,2], Klaus T. Preissner [4] and Elisabeth Deindl [1,2,*]

[1] Institute of Surgical Research at the Walter Brendel Centre of Experimental Medicine, University Hospital, Ludwig-Maximilians-Universität München, 81377 Munich, Germany; amanda.zhu@med.uni-muenchen.de (A.Z.); carolin.baur@med.uni-muenchen.de (C.B.); p.goetz@med.uni-muenchen.de (P.G.); katharina.elbs@med.uni-muenchen.de (K.E.); manuel.lasch@med.uni-muenchen.de (M.L.); anna.braumandl@web.de (A.F.)
[2] Biomedical Center, Institute of Cardiovascular Physiology and Pathophysiology, Faculty of Medicine, Ludwig-Maximilians-Universität München, 82152 Planegg-Martinsried, Germany
[3] Department of Otorhinolaryngology, Head and Neck Surgery, University Hospital, Ludwig-Maximilians-Universität München, 81377 Munich, Germany
[4] Department of Cardiology, Kerckhoff-Heart Research Institute, Faculty of Medicine, Justus-Liebig-University, 35392 Giessen, Germany; klaus.t.preissner@biochemie.med.uni-giessen.de
* Correspondence: elisabeth.deindl@med.uni-muenchen.de; Tel.: +49-(0)-89–2180–76504

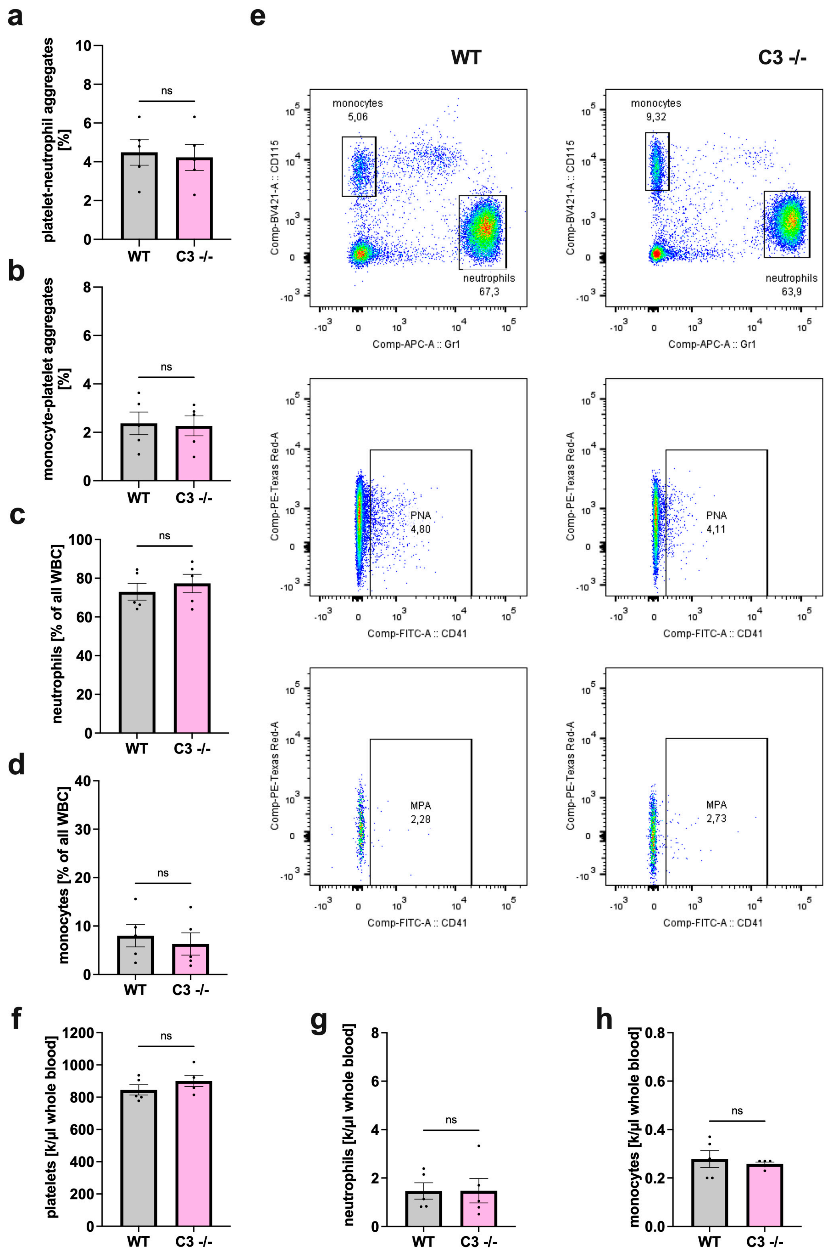

Figure S1. C3 has no significant impact on platelet-neutrophil aggregates (PNA) and monocyte-platelet aggregates (MPA) formation after femoral artery occlusion. The scatter plots with bars display flow cytometry analyses of (**a**) the percentage of PNAs relative to the total number of neutrophils, (**b**) the percentage of MPAs relative to the total number of monocytes, (**c**) the percentage of neutrophils and (**d**) monocytes relative to the number of all counted white blood cells of wildtype (WT) and C3 -/- mice 24 hours after femoral artery ligation (FAL). Platelets were detected by an anti-CD41 (FITC) antibody, neutrophils were identified by anti-CD11b (PE) and anti-Gr-1 (APC) antibodies and monocytes by anti-CD11b (PE) and anti-CD115 (Brilliant Violet (BV) 421) antibodies. (**e**) Representative pictures showing the applied gating strategy. The scatter plots with bars below show the number of (**f**) platelets, (**g**) neutrophils and (**h**) monocytes per microliter blood measured by differential blood count of WT and C3 -/- mice 24 h after FAL. Data shown are means ± SEM, $n = 5$ mice per group, not significant (ns): $p \geq 0.05$, WT were compared to C3 -/- mice by unpaired student's t-test.

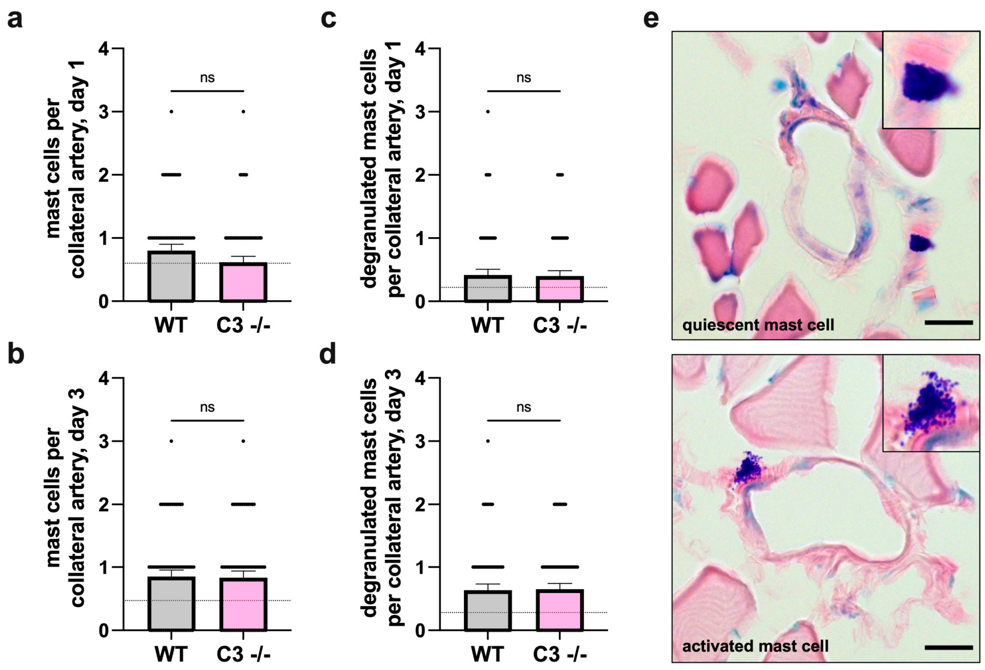

Figure S2. C3 has no impact on mast cell recruitment and activation 24 h and 3 days after induction of arteriogenesis. The scatter plots with bars describe the total number of perivascular mast cells of wildtype (WT) and C3 -/- mice per growing collateral (**a**) 24 h and (**b**) 3 days after femoral artery ligation (FAL), as well as the number of degranulated mast cells of WT and C3 -/- mice per collateral artery (**c**) 24 h and (**d**) 3 days after FAL. The dashed horizontal line in (a-d) represents the mean sham value. Data shown are means ± SEM, $n = 6$ mice per group with 5 slices, 2 collaterals each, ns ≥ 0.05. WT were compared to C3 -/- mice by unpaired student's t-test. (**e**) Representative Giemsa stains of evaluated tissue samples showing a quiescent mast cell (upper image) and an activated mast cell (lower image). Insert in the upper left corner displays a magnification of the mast cell. Scale bar: 20 μm.

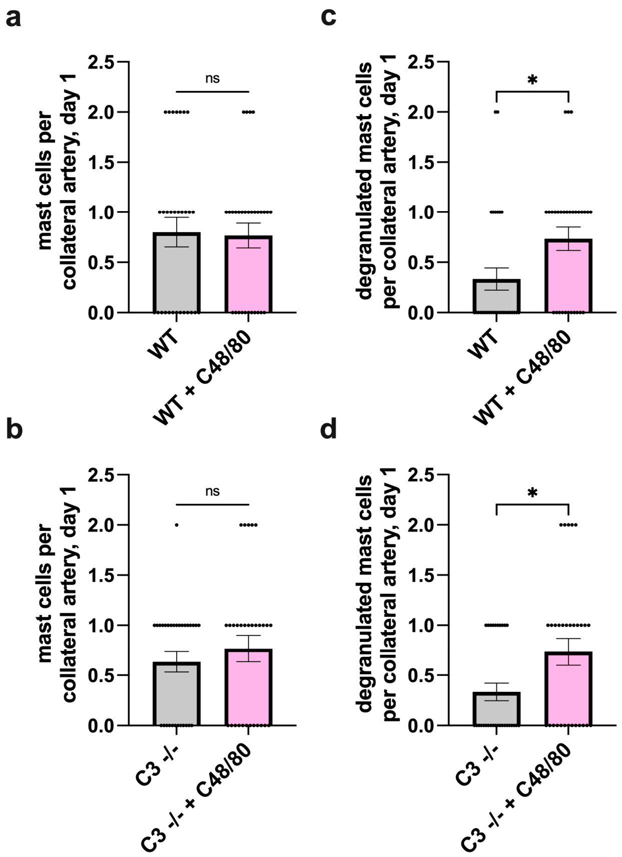

Figure S3. C48/80 leads to mast cell degranulation in wildtype (WT) and C3 -/- mice. Scatter plots with bars representing the total number of mast cells in (**a**) WT and WT after treatment with C48/80 or in (**b**) C3 -/- and C3 -/- after treatment with C48/80 and the number of degranulated mast cells of (**c**) WT and WT after treatment with C48/80 or (**d**) C3 -/- and C3 -/- after treatment with C48/80 24 h after femoral artery ligation (FAL) per growing collateral artery. Data shown are means ± SEM, n = 3 mice per group with 5 slices, 2 collaterals each, * $p < 0.05$ and ns ≥ 0.05, unpaired student's t-test.

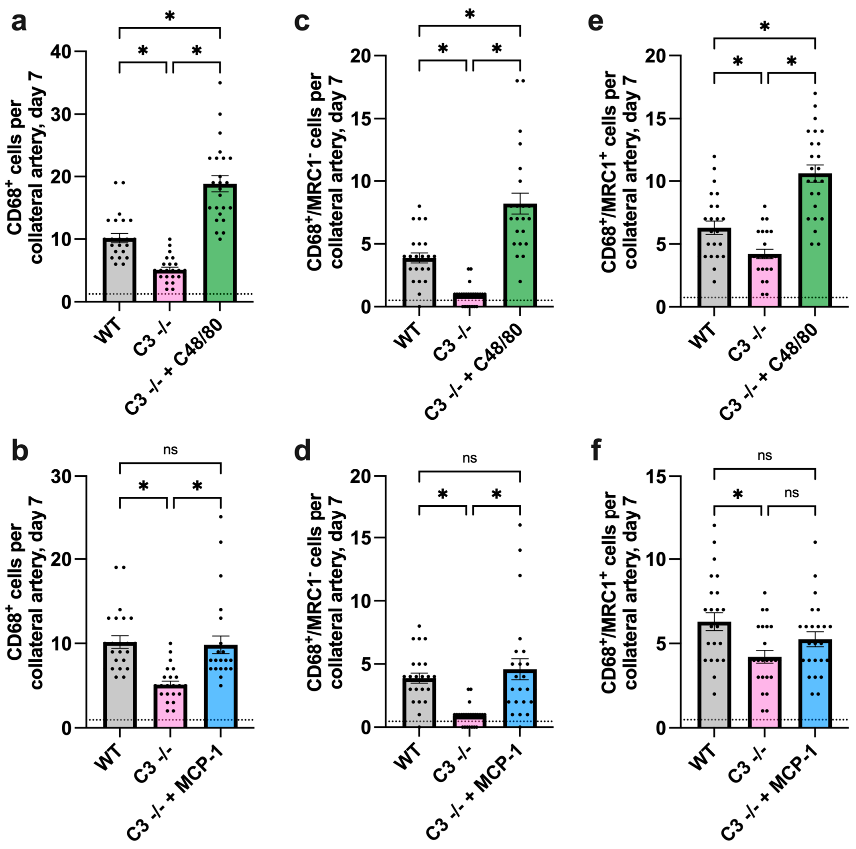

Figure S4. Exposition towards C48/80 or MCP-1 elevates the number of perivascular M1-like macrophages in C3 -/- mice 7 days after induction of arteriogenesis. The scatter plots with bars display (**a**) the total number of perivascular macrophages ($CD68^{+}$ cells) of wildtype (WT, grey bars), C3 -/- (pink bars) and C3 -/- after treatment with C48/80 (green bars) or (**b**) MCP-1 (blue bars) 7 days after femoral artery ligation (FAL), as well as the number of M1-like macrophages ($CD68^{+}/MRC1^{-}$ cells) after treatment with (**c**) C48/80 or (**d**) MCP-1 and the number of M2-like macrophages ($CD68^{+}/MRC1^{+}$ cells) after treatment with (**e**) C48/80 or (**f**) MCP-1 per collateral artery on day 7 after induction of arteriogenesis. The dashed horizontal line represents the mean sham value. Data shown are means ± SEM, n = 4 mice per group with 3 slices, 2 collaterals each, * $p < 0.05$ and ns ≥ 0.05, compared by one-way ANOVA with Bonferroni's multiple comparison test.
